# Complex regional pain syndrome with associated chest wall dystonia: a case report

**DOI:** 10.1186/1749-7221-6-6

**Published:** 2011-09-26

**Authors:** David J Irwin, Robert J Schwartzman

**Affiliations:** 1 Drexel University College of Medicine, Department of Neurology, Philadelphia, PA, USA

**Keywords:** complex regional pain syndrome, dystonia, movement disorder, dyspnea

## Abstract

Patients with complex regional pain syndrome (CRPS) often suffer from an array of associated movement disorders, including dystonia of an affected limb. We present a case of a patient with long standing CRPS after a brachial plexus injury, who after displaying several features of the movement disorder previously, developed painful dystonia of chest wall musculature. Detailed neurologic examination found palpable sustained contractions of the pectoral and intercostal muscles in addition to surface allodynia. Needle electromyography of the intercostal and paraspinal muscles supported the diagnosis of dystonia. In addition, pulmonary function testing showed both restrictive and obstructive features in the absence of a clear cardiopulmonary etiology. Treatment was initiated with intrathecal baclofen and the patient had symptomatic relief and improvement of dystonia. This case illustrates a novel form of the movement disorder associated with CRPS with response to intrathecal baclofen treatment.

## Background

Complex regional pain syndrome (CRPS) is most often caused by a fracture or soft tissue injury of an extremity or a surgical procedure [1]. Factor analysis demonstrates that signs and symptoms of the syndrome cluster into four subgroups: 1) abnormalities in pain processing that cause allodynia, hyperalgesia and hyperpathia; 2) skin color and temperature change; 3) neurogenic edema, vasomotor and sudomotor abnormalities; and 4) a movement disorder and trophic changes [2]. The movement disorder is manifest as a combination of difficulty initiating and maintaining movement, weakness, postural and intention tremor, myoclonus, spasm, increased tone, abnormalities of reaching and grasping and dystonia [3,4]. Dystonia in CRPS is most likely a peripherally induced, focal dystonia [5].

In one study approximately 62% of CRPS patients were found to have an associated movement disorder, with dystonia being the most common [6]. Dystonia in CRPS patients is most common in the affected limb, with adduction of the arm and flexion of wrist and fingers in the upper extremity and internal rotation of the hip with plantar flexion and inversion of the foot in the lower extremity [3]. The presence of dystonia in CRPS patients is associated with longer disease duration and a younger age [6]. The onset of the movement disorder is variable but may precede other manifestations of the disease, and can occur five years or longer after disease onset [3,6].

The presence of dystonia in one extremity increases the risk of dystonia in a second extremity [6], with ipsilateral spread the most common pattern [3]. Generalized forms of dystonia can occur that involve all limbs [7,8]; however dystonia of axial muscles (intercostal, pectoralis and oblique muscles) that causes dyspnea has not been reported.

## Case Presentation

The patient is a 51-year-old female who has been followed in neurologic consultation by the author (RJS) since 1987 for her chronic regional pain syndrome. She first presented with a brachial plexus traction injury after a fall. Pain symptoms progressed over the next two years to include total body burning and lancinating pains. At this point she had all factors for diagnosis of CRPS [9].

On physical examination the patient was moderately obese with an anteroflexed body posture and increased carrying angles of the arms. She had a paucity of spontaneous movement. Sensory examination showed severe generalized dynamic and static mechano allodynia and loss of surround inhibition to pinprick and a cold stimulus. In addition, she had severe generalized deep muscle sensitization and joint pain. She had hyperalgesia to pinprick and "wind up" as well as cold allodynia in all quadrants of her body. She had longstanding chest pain in the distribution of the intercostobrachial nerve [10]. Autonomic involvement was demonstrated by cold extremities and generalized hyperhidrosis and was associated with moderate neurogenic edema in the lower extremities and livedo reticularis of the skin. Dystonic posturing of the lower extremity was noted early during a few visits and was evidenced by internal rotation of the hip and plantar flexion and inversion of the foot. In addition to overt dystonia, the patient developed ambulatory dysfunction due to weakness and difficulty initiating movements. On one occasion she noted her legs did "not feel like hers." She had difficulty initiating and maintaining fine movements in all extremities. On one visit a postural and intention tremor of the hands and head was noted.

She had failed numerous surgical and medical interventions and developed opiate dependency requiring high doses of intrathecal dilaudid via a subcutaneous pump. Other medical problems included adrenal insufficiency, obstructive sleep apnea that required maintenance on nocturnal bi-level positive airway pressure, chronic gastroparesis, chronic elevation of the right hemi-diaphragm, and hypothyroidism.

Approximately twenty-four years after the onset of her CRPS, the patient began having respiratory symptoms of dyspnea on exertion. Palpation of the chest wall showed restrictive chest expansion and sustained contractions of the intercostal musculature, consistent with dystonia. Electromyography (EMG) of the transverses thoraces muscles and paraspinal muscles at T6 and T10 showed normal insertional activity and motor unit morphology. During neurophysiologic testing there was an inability to relax these muscles voluntarily by the patient.

Initial pulmonary function testing showed a mixed restrictive and obstructive picture with a response to bronchodilators (Table 1). Spirometry study met American thoracic society criteria for acceptability [11], with the exception of forced vital capacity on the first study due to fatigue. CT scanning of the chest did not display any features of interstitial lung disease. Echocardiogram showed grade I diastolic dysfunction with no structural heart, valve disease, or pulmonary hypertension.

**Table 1:** Pulmonary function testing results before and after intrathecal baclofen treatment

	FVC	FEV1	FEF 25-75%	PEF	TLC	RV	DLCO
**Study 1**† **Pre-Bronchodilator**	1.83 L (44% Ref)	1.60 L (49% Ref)	2.20 L/sec (73% Ref)	5.34 L/sec (71% Ref)	2.71 L (45% Ref)	0.88 L (40% Ref)	12.20 mL/mmHg/min (44% Ref)
**Post -Bronchodilator**	2.06 L (50% Ref)	1.78 L (54% Ref)	2.44 L/sec (81% Ref)	4.62 L/sec (62% Ref)	NA	NA	NA
**Study 2**†† **Pre-Bronchodialtor**	1.95 L (47% Ref)	1.57 L (49% Ref)	1.59 L/sec (53% Ref)	4.74 L/sec (64% Ref)	3.07 L (52% Ref)	1.10 L (50% Ref)	11.00 mL/mmHg/min (36% Ref)
**Post -Bronchodilator**	2.18 L (53% Ref)	1.79 L (55% Ref)	1.97 L/sec (66% Ref)	4.89 L/sec (66% Ref)	NA	NA	NA

† Patient intrathecal medication dose at time of study: Baclofen 0 μg/day, Dilaudid 26.5 mg/day

†† Patient intrathecal medication dose at time of study: Baclofen 400 μg/day, Dilaudid 21.5 mg/day

After receiving a five-day continuous intravenous sub-anesthetic dose of ketamine by infusion (40 mg/hr; midazolam 4 mg/6 hr; 0.1 mg of clonidine), her intrathecal dilaudid was gradually weaned from 79 mg/day to 21 mg/day. In addition, intrathecal baclofen was added at an initial dose of 75 μg/day. Baclofen was up titrated by approximately 50 μg/day every two weeks to a final dose of 600 μg/day. The patient experienced symptomatic relief of her chest wall discomfort and dyspnea beginning at doses of 125 μg of baclofen per day. Chest wall dystonia was much improved on serial neurologic examinations. Pulmonary function testing results were not significantly changed after baclofen treatment (Table 1). She was maintained at a dose of 600 μg/day of intrathecal baclofen and has been asymptomatic in regard to lethargy, weakness, nausea, headaches, or psychosis.

## Conclusions

To our knowledge, this is the first report of dystonia of the chest wall musculature associated with CRPS. The electrodiagnostic evidence of dystonia was limited to patient-dependent factors, as the EMG testing showed normal motor unit activation and morphology. It should be noted that the EMG was performed while the patient was receiving 425 μg/day of intrathecal baclofen, which could affect motor unit activation. A neurophysiologic study of dystonia in CRPS patients found a similar inability to alter muscle activity voluntarily, in addition to decreased inhibition from activation of antagonist muscle groups [12]. One could argue that the observed inability to relax the chest was musculature was psychogenic; however, voluntary sustained contraction of these axial muscle groups would be very difficult to perform compared to an extremity. In addition, this patient did not display pseudoneurological signs others have argued to be present in CRPS [13].

The significance of the chest wall dystonia in regards to pulmonary symptoms is unclear, but most likely contributes in part to the restrictive pulmonary function pattern observed. Patients with idiopathic and secondary dystonia have been noted to have excessive contractions of the diaphragm and upper airways contributing to symptoms of dyspnea [14]. This patient also had obstructive features and a response to bronchodilators, which most likely represents concomitant asthmatic disease. There was no intrinsic disease of the lung parenchyma on CT scanning that could be responsible for her obstructive and restrictive pulmonary function. Vocal cord involvement as a cause for her dyspnea was also unlikely, as there was no dysphonia. Her body habitus, chest wall pain, and paralysis of the right hemi-diaphragm can also contribute to restrictive lung disease. The diaphragm paralysis in this patient is most likely another manifestation of dystonia as spasmodic contraction of the diaphragm may be seen in dystonic patients [14].

The mechanism of dystonia in CRPS is not completely understood but is generally thought to involve neural circuits that mediate sensory-motor integration [15-17]. Recent studies demonstrate impaired inhibition both at cortical and spinal cord levels [18-20]. Present evidence suggests that a major component of the mechanism of dystonia in CRPS involves disinhibition of painful nociceptive withdrawal reflexes in the spinal cord [6]. These reflexes are initiated by activity in C and A-delta primary pain fibers that colocalize vasoactive neuropeptides with glutamate and are also pivotal in neurogenic inflammation [21]. Substance P is released from pain afferents and activates NK1 receptors on lamina I neurons of the dorsal horn that is important in the induction of long term potentiation of these pain transmission neurons [22]. Evidence of spinal cord inflammation has been demonstrated in CRPS patients who have increased levels of inflammatory cytokines in their spinal fluid [23,24]. Pathologic examination of a severe longstanding CRPS patient has shown microglial and astrocytic activation most prominent at the segmental level of injury, but also as a gradient spread throughout the spinal cord bilaterally [25]. It is possible that this inflammatory activation of the spinal cord resulted in dystonia of the axial musculature through disinhibition of GABAergic inhibitory neurons of the dorsal horn at thoracic levels mediated by SP and inflammatory cytokines. The presented patient has suffered with CRPS for over twenty years and the one autopsied had a six year course suggesting that axial dystonia is a late manifestation of the syndrome.

Intrathecal baclofen is effective in treatment of the limb dystonia of CRPS at a mean dose of 415 μg/day [26]. Our patient responded at 600 μg/day. Symptomatic improvement from intrathecal baclofen implicates spinal cord involvement in this form of dystonia. Its greatest concentration is in the dorsal horn of the spinal cord in primary afferent fibers [27]. The axial dystonia seen in this patient suggests a link between immune mediated cytokine release and substance P activation of nocifensor reflexes in the thoracic cord in the axial dystonia of CRPS.

## Consent

Written informed consent was obtained from the patient for publication of this case report. A copy of the written consent is available for review by the Editor-in-Chief of this journal

## Competing interests

The authors declare that they have no competing interests.

## Authors' contributions

RJS formulated the project. DI and RJS contributed in taking the patient history, physical exam, and preparation of the manuscript. All authors read and approved the final manuscript.

## References

[B1] Schwartzman RJ Patel M Grothusen JR Alexander GM Efficacy of 5-day continuous lidocaine infusion for the treatment of refractory complex regional pain syndrome Pain Med 2009 6 2 401 412 10.1111/j.1526-4637.2009.00573.x 19284488

[B2] Harden RN Bruehl S Wilson, PR, Stanton-Hicks, MD, Harden, RN Diagnostic criteria: the statistical derivation of the four criterion factors CRPS: Current Diagnosis and Therapy 2005 Seattle: IASP Press 45 58

[B3] Schwartzman RJ Kerrigan J The movement disorder of reflex sympathetic dystrophy Neurology 1990 6 1 57 61 2296383 10.1212/wnl.40.1.57

[B4] Van Hilten JJ Blumberg HPD Schwartzman RJ Wilson, PR, Stanton-Hicks, MD, Harden, RN Factor IV: Movement disorders and dystrophy: clinical and pathophysiological aspects CRPS: Current Diagnosis and Therapy 2005 Seattle: IASP Press 45 58

[B5] Schott GD Peripherally-triggered CRPS and dystonia Pain 2007 6 203 207 10.1016/j.pain.2007.04.013 17512665

[B6] van Rijn MA Marinus J Putter H van Hilten JJ Onset and progression of dystonia in complex regional pain syndrome Pain 2007 6 3 287 293 10.1016/j.pain.2007.03.027 17499924

[B7] van Hilten JJ van de Beek WJ Roep BO Multifocal or generalized dystonia in complex regional pain syndrome: a distinct clinical entity associated with HLA-DR 13 Ann Neurol 2000 6 113 116 10.1002/1531-8249(200007)48:1<113::AID-ANA18>3.0.CO;2-9 10894225

[B8] van Hilten JJ van de Beek WJ Vein AA van Dijk JG Middelkoop HA Clinical aspects of multifocal or generalized tonic dystonia in reflex sympathetic dystrophy Neurol 2001 6 1762 1765 10.1212/wnl.56.12.1762 11425951

[B9] Harden RN Bruehl S Stanton-Hicks M Wilson PR Proposed new diagnostic criteria for complex regional pain syndrome Pain med 2007 6 326 331 10.1111/j.1526-4637.2006.00169.x 17610454

[B10] Rasmussen J Grothusen JR Rosso AL Schwartzman RJ Atypical chest pain: evidence of intercostobrachial nerve sensitization in complex regional pain syndrome Pain Phys 2009 6 e329 e324 19787018

[B11] Standardization of spirometry- 1987 update Official statement of the American Thoracic Society Respir Care 1987 6 11 1039 1060 10315742

[B12] van de Beek WJ Vein A Hilgevoord AA van Dijk JG van Hilten BJ Neurophysiologic aspects of patients with generalized or multifocal tonic dystonia of reflex sympathetic dystrophy J Clin Neurophysiol 2002 6 1 77 83 10.1097/00004691-200201000-00011 11896357

[B13] Verdugo R Ochoa JL Abnormal movements in complex regional pain syndrome: assessment of their nature Muscle Nerve 2000 6 198 205 10.1002/(SICI)1097-4598(200002)23:2<198::AID-MUS9>3.0.CO;2-4 10639611

[B14] Braun N Abd A Baer J Blitzer A Stewart C Brin M Dyspnea in dystonia: a functional evaluation Chest 1995 6 5 1309 1316 10.1378/chest.107.5.1309 7750324

[B15] Mink JW Abnormal circuit function in dystonia Neurol 2006 6 7 959 10.1212/01.wnl.0000215937.63781.9a 16610101

[B16] Huang YZ Trender-Gerhard I Edwards MJ Motor system inhibition in dopa-responsive dystonia and its modulation by treatment Neurol 2006 6 7 1088 1090 10.1212/01.wnl.0000214304.03105.f4 16606922

[B17] Tisch S Limousin P Rothwell JC Changes in forearm reciprocal inhibition following pallidal stimulation for dystonia Neurol 2006 6 7 1091 1093 10.1212/01.wnl.0000204649.36458.8f 16606923

[B18] van de Beek WJ Vein A Hilgevoord AJ van Dijk G van Hilten B Neurophysiologic aspects of patients with generalized or multifocal tonic dystonia of reflex sympathetic dystrophy J Clin Neurophysiol 2002 6 1 77 83 10.1097/00004691-200201000-00011 11896357

[B19] Schouten AC Van de Beek WJ Van Hilten JJ Van der Helm FC Proprioceptive reflexes in patients with reflex sympathetic dystrophy Exp Brain Res 2003 6 1 1 8 10.1007/s00221-003-1420-x 12743675

[B20] Schwenkreis P Janssen F Rommel O Bilateral motor cortex disinhibition in complex regional pain syndrome (CRPS) type I of the hand Neurol 2003 6 4 515 519 10.1212/wnl.61.4.515 12939426

[B21] Weber M Birklein F Neundorfer B Schmelz M Facilitated neurogenic inflammation in complex regional pain syndrome Pain 2001 6 3 251 257 10.1016/S0304-3959(00)00445-0 11275381

[B22] Schouenborg J Learning in sensorimotor circuits Curr Opin Neurobiol 2004 6 6 693 697 10.1016/j.conb.2004.10.009 15582370

[B23] Alexander GM van Rijn MA van Hilten JJ Perreault MJ Schwartzman RJ Changes in cerebrospinal fluid levels of pro-inflammatory cytokines in CRPS Pain 2005 6 213 219 10.1016/j.pain.2005.04.013 15964681

[B24] Alexander GM Perreault MJ Reichenberger ER Schwartzman RJ Changes in immune and glial markers in the CSF of patients with complex regional pain syndrome Brain Behav. Immun 2007 6 668 676 10.1016/j.bbi.2006.10.009 17129705

[B25] Del Valle L Schwartzman RJ Alexander G Spinal cord histopathological alterations in a patient with complex regional pain syndrome Brain Behav. Immunity 2009 6 85 91 10.1016/j.bbi.2008.08.004 18786633

[B26] van Rijn MA Munts AG Marinus J Voormolen JHC de Boer KS Teepe-Twiss IM van Dasselaar NT Delhaas EM van Hilten JJ Intrathecal baclofen for dystonia of complex regional pain syndrome Pain 2009 6 41 47 10.1016/j.pain.2009.01.014 19232828

[B27] Malcangio M Bowery NG GABA and its receptors in the spinal cord Trends Pharmacol Sci 1996 6 457 462 10.1016/S0165-6147(96)01013-9 9014500

